# Extracurricular Physical Activities and the Condition of Being an Only Child as a Conditioning Factor in the Psychomotor Development of 5-Year-Old Children

**DOI:** 10.3389/fped.2021.684418

**Published:** 2021-07-05

**Authors:** Pedro Gil-Madrona, Sonia J. Romero-Martínez, Carmen C. Roz-Faraco

**Affiliations:** ^1^Department of Didactics on Physical, Artistic and Music Education, Faculty of Education, Albacete, University of Castilla La Mancha, Ciudad Real, Spain; ^2^Department of Methodology of the Behavioral Sciences, Faculty of Psychology, National University of Distance Education (UNED), Madrid, Spain; ^3^Degree in Early Childhood Education and Primary Education, Faculty of Education, International University of la Rioja, Madrid, Spain

**Keywords:** psychomotor development, infant education, physical education, extracurricular activities, only children

## Abstract

In early childhood education, there is a great interdependence among motor, affective, and cognitive development. A better understanding of psychomotor development and its variables by pediatricians and those who oversee educational tasks at this stage of development, such as teachers, psychologists, counselors, and parents themselves, can influence the design of educational intervention proposals. To that effect, the present study aims to analyze the influence of some family characteristics such as the condition of being an only child or having siblings and whether the child carries out extracurricular activities linked to physical activities and sports. To achieve this objective, a Checklist of Psychomotor Activities (CPA) assessment instrument was used based on the observation of a sample of 694 children aged 5 years who were enrolled in the second cycle of child education in the province of Albacete. The results show that those children who performed extracurricular activities related to physical activities and sports had a greater development of laterality and postural tonic control than those who did not attend this type of activities. At the same time, differences were found in the affective and relational levels and in the perceptual-motor aspects in favor of children who have siblings compared with children who are only children. There is no other research that addresses both issues in the same study, and this is a strength of the present study.

## Introduction

Do the decisions of the parents significantly influence the development of children? This study aims to delve into two aspects that can answer this question, the number of siblings that have the children, and the type of after-school activities the children will do outside the school and how they influence the psychomotor development of 5-year-old children who are in the second cycle of early childhood education of the community of Albacete (Spain).

In the stage of early childhood education, the integral development of students is very important, because it is one of the main objectives of this educational stage. Physical education, motor activity, and psychomotricity are aspects that correlate and directly influence perceptual-motor, physical-motor, cognitive, and effective-relational development (1–4) being an important aspect in childhood inside and outside the school grounds. To know the effects of some variables on this area provides the educators tools for the improvement of psychomotor skills of children in the early stages of primary education.

Motor skills, in its systematic form, physical education, or psychomotricity in early childhood education is one of the subjects or areas that develops these aspects; however, children who carry out extracurricular activities of a sporting nature have an extra motor benefit, of which they only carry out physical activities in schools (5, 6). In turn, it is a decisive component in the parenting style, giving their children participation in extracurricular sports activities (ESAs), (7).

The benefits of ESAs carried out by children at this educational stage have a great impact on the physical, social, emotional, and psychological health of children expressed by various authors (8–10) who similarly believe that extracurricular activities facilitate the development of non-cognitive skills.

Another important variable in this research is the difference in relational psychomotor and affective skills between children who have siblings and those who do not. Authors like Berger and Nuzzo (11) have determined that children who have older siblings achieve motor skills before those older siblings; however, in number, they develop more skills and acquire these faster.

Due to the importance of the mentioned variables, the present research analyses the relevance of ESAs in the development of motor, perceptual, and affective skills and if there are differences in these skills between children who have siblings and those who have not. Next, previous studies on the two variables under study will be presented.

### Extracurricular Sports Activities and Their Impact on Motor Development in Children's Education

Motor stimulation not only can benefit the physical development of 5-year-olds but in turn brings positive skills in cognitive and affective factors, since good motor development has a direct impact on perceptual, physical, motor, cognitive, and affective faculties. The work of motor patterns in early childhood is the basis for the realization of basic, as well as specialized tasks that are present in daily activities (12, 13). Motor development is characterized by being a process that is carried out throughout the development of the infant, composed of qualitative and quantitative aspects (7, 14, 15). ESAs bring cognitive benefits that are closely related to psychomotor aspects such as laterality, balance, spatial notion, perception, and postural tonic control. There are several studies that corroborate this relationship, for example.

Rasberry et al. (16) found 251 positive associations in the analysis of 43 previous studies that analyze the benefit of ESAs and its relationship to the cognitive aspects expressed through academic performance, academic behavior, cognitive skills, and student attitudes. Another reference that corroborates the benefit of the ESAs is the study carried out by Temple et al. (17), who corroborated that the most active after-school activities were associated with a higher level of motor skills. And finally, the study developed by Raudsepp and Päll (18) confirmed that performing specific activities significantly improved object control skills.

### Relational Affective Factors Implicit in Extracurricular Sports Activities

The factors that influence the socialization process that are implicit in the ESAs (such as the affective factors and relational, motivational, and experiences of pleasure of acting and performing a sports activity that leads to socialization with family, colleagues, sports coaches, and teachers) bring with them not only emotional but psychological benefits as posed in the study of Bailey et al. (19). In turn, participating in an active and consenting way of expressing skills through its execution improves the social integration and brings emotional and psychomotor benefits (20).

According to Sanmartín (21), sports is recognized as an inclusive space, which will provide the individual with benefits in its psychological and social aspects. As we see, there is a conviction that habits established in the early ages will engage them significantly, marking a continuity of this style in adulthood, and also that if active lifestyles accompany healthy lifestyles, we will be able to enhance them through physical activities and sports in the early stages of childhood by encouraging the development of potentially healthy (mentally and physically) individuals.

### Differences in Motor Development Between Children Who Have Siblings or Not

The children's brain is constantly learning and is highly perceptive of environmental information through movement and exploration. Related to it, is relevant to ask: is there really a difference in the motor development between children who have siblings or those who do not have siblings?

To answer this question, it is important to take into consideration that the child psychomotricity development is mediated by the environment but also this process is related to its physical constitution, namely, there are basic and egalitarian aspects in each individual; however, the speed of information processing will be different for those children who possess imitative behavior of an equal that stimulates it, not only from the motor, psychomotor, or perceptive point of view but also stimulating the various emotions that can be fostered through peer-to-peer play (22). In the same line, the order of birth in a family is also a factor that can affect the development of motor capacity.

Children with older siblings have better motor performance than only or first-born children (11, 23). It is found that one of the typical sequences is that an older child initially performs a task while the younger siblings watch or spend a lot of time observing the performance of the older sibling and replicating his/her movements (24). Older siblings provide more advanced development models for younger siblings and help to create an enriched and stimulating environment that seems to improve the development of younger siblings (22, 25). More recently, Giagazoglou et al. (26) analyzed motor development in light of the order of birth occupied by the child and concluded that this order did not have an influence on motor development.

### Relational Affective Factors in Children Who Have Siblings or Not

From a psychomotor and affective-relational point of view, most studies establish that there is a significant difference between children who have older siblings and those who are an only child; from a playful point of view in motor activities, the older sibling impersonates a figure of secondary attachment, in the presence of which the younger sibling can explore the context more safely since he/she feels being accompanied and safe with him/her, or the older siblings diminish the fear of strangers, becoming a significant fraternal interaction (27). However, other studies, such as Poston and Falbo (28), mentioned that affective-relational differences are in balance between children with or without siblings of Chinese origin and in some case in some variables slightly higher in children who have siblings.

## Method

### Participants

Information has been obtained from 694 5-year-olds schooled in the third cycle of early childhood education. Information has been collected and provided by teachers from 32 child education groups, from the towns of Almansa, La Roda, Albacete, Chinchilla, Aguas Nuevas, Alcázar de San Juan, Villarobledo, Casas Ibáñez, Riópar, Munera, and Quintanar del Rey. The sample has been non-probabilistic in nature; the sampling technique has been used on purpose, as voluntary collaboration has been requested in all schools in Albacete province, and we have finally worked with the schools that agreed to participate.

The sample consists of 46.7% girls and 53.3% boys. Most participants belong to public schools 69.5 vs. 30.5% who are students of concerted schools. It should be noted that 65% of children practice some extracurricular activities of a sporting type and 81.4% have siblings.

### Instruments

The Checklist of Psychomotor Activities (CPA) was used to measure children's psychomotor development. The CPA test consists of three scales:

- Scale of Psycho-Motor Aspects (SPMA), composed of five factors or dimensions: Laterality (LAT; seven items), Dynamic Coordination (DC; six items), Tonic-Postural Control (CTP; three items), Motor Execution (ME; three items), and Balance (BAL; five items).
- Perceptual Aspects Scale (PAS), composed of five factors or dimensions: Respiratory Control (RC; three items), Body Image (BI; four items), Motor Dissociation (MD; three items), Visuo-Motor Coordination (VMC; six items), and Spatial Orientation (SO; two items).
- Scale of Socio-Emotional Aspects (SSEA), composed of two factors or dimensions: Emotional Control (EC; six items) and Social Relations (SR; five items).

Participants were evaluated by their teachers using a 5-point Likert scale from 1 (never) to 5 (always), depending on their ability to perform the proposed task on each item. The CPA has been shown to have adequate psychometric properties with good reliability (Cronbach's alpha) ranging from 0.572 (laterality) to 0.872 (balance) on the SPMA scale, between 0.514 (spatial orientation) and 0.825 (respiratory control) on the PAS scale, and between 0.572 (emotional control) and 0.800 (social relations) on the SSEA scale. Reliability considering the entire scale is remarkably high: 0.935. The authors also present evidence of factorial, content, and discriminatory validity.

### Procedure

First, different schools in the province of Albacete, Spain, were contacted. An explanatory document with the objectives of the research was presented to the school's directors, and their voluntary participation was requested. Second, informed consent was requested from parents in schools that eventually agreed to participate; parents also answered a questionnaire asking about the other study variables: whether they have siblings and whether they do ESAs. Third, physical education teachers were training to use the CPA instrument. Fourth, the teachers in each course did the evaluation of the children with the help of a member of the research team, during an hour of physical education class. Finally, CPA evaluation data and parent questionnaires were combined into a database that was used for analysis.

### Data Design and Analysis

A quantitative, non-experimental, descriptive, and explanatory cross-cutting study was carried out. It is also known as simple prospective *ex post facto* design.

### Variables

The variables that have been considered to establish the comparison are family members (if you are an only child or otherwise have siblings) and education (if you do ESAs) of the participating children.

Data analyses include two parts: the first part contains the description of the variables according to their frequency and percentage distribution; the second part contains the analysis of the differences between groups that has been carried out through the non-parametric U tests of Mann–Whitney and Kruskal–Wallis.

## Results

### Description of the Variables Analyzed

Tables 1–3 present the distribution of the variables studied. Table 1 shows that 65% of the sample have at least one sibling, while only 19% do not have siblings. On the other hand, in Table 2, it can also be observed that most children (again 65%) practice sports school activities. For its part, in Table 3, it is appreciated that as the number of siblings increases, the foray of students in ESAs, being only children with 52%, the least favored in this range of 0–4 siblings, however, it can be seen that 52% in front of the students have siblings or do not benefit from the relational affective stimulation that the extracurricular activities gives.

**Table 1:** Frequency distribution and number of siblings.

***N***	**Frequencies**	**% of total**	**% accumulated**
0	132	19.0%	19.0%
1	319	46.0%	65.1%
2	195	28.1%	93.2%
3	35	5.1%	98.3%
4	10	1.4%	99.7%
6	1	0.1%	99.9%
7	1	0.1%	100.0%

**Table 2:** Frequency distribution of after-school sports activities.

**After-school activities**	**Frequency**	**% of total**	**% accumulated**
No	250	36.0%	36.0%
Yes	444	64.0%	100.0%

**Table 3:** Practice of extracurricular activities according to the number of siblings.

		**Practice of sports extracurricular activities**	
		**No (%)**	**Yes (%)**
Number of siblings	0	48	52
	1	34	66
	2	28	72
	3	31	69
	4	40	60
	6	100	0
	7	100	0

### Differences Depending on the Practice of Extracurricular Sports Activities

The Mann–Whitney *U*-test results according to the practice of after-school sports activities in the physical-motor aspects are presented in Table 4. It is observed that there are significant differences in laterality and postural tonic control, with better performance (higher average ranges) in children who practice some extracurricular activities of sports type.

**Table 4:** Mann–Whitney-test and descriptive statistics according to the practice of sports activities on the physical-motor aspects.

	**Mann–Whitney-test**					**Descriptive statistics**				
**Var**	***U***	***Z***	***p*-value**	***R***	**Practice**	**Mean (SD)**	**Me**	**Range**	**G1**	**G2**
LT	41,520.0	−4.250	0.000	0.167 (1)	No	25.81 (4.62)	23	294.19	0.872	−0.669
					Yes	27.42 (5.06)	27	360.27	0.043	−1.038
DC	48,986.0	−1.139	0.255	0.044	No	26.95 (3.35)	28	325.69	−1.197	0.956
					Yes	27.14 (3.22)	28	343.15	−1.193	1.189
ME	50,554.5	−0.495	0.620	0.019	No	13.83 (1.53)	15	341.69	−1.466	2.214
					Yes	13.74 (1.68)	14	334.45	−1.751	3.497
TPC	43,346.0	−3.546	0.000	0.139 (1)	No	12.59 (2.22)	13	301.89	−0.683	−0.305
					Yes	13.12 (3.07)	14	356.08	−1.231	1.881
BAL	51,440.5	−0.096	0.924	0.000	No	21.57 (3.45)	22.5	336.05	−0.628	−0.710
					Yes	21.41 (3.55)	22	337.52	−0.907	0.747
SUM	47,055.5	−1.916	0.055	0.075	No	100.75 (10.69)	103	336.05	−0.705	0.358
					Yes	102.83 (12.23)	103.5	337.52	−1.057	2.687

*Significant differences p < 0.05*.

*SD, standard deviation; Me, Median; G1, skewness; G2, kurtosis; LT, laterality; DC, dynamic coordination; ME, motor execution; TPC, tonic-postural control; BAL, balance; SUM, sum of all items*.

Table 5 presents the results of the Mann–Whitney-test and descriptive statistics on the perceptual-motor aspects according to the practice of extracurricular activities of sports type. In the table, it can be observed that there are only significant differences in spatial orientation, these being favorable to the group that practices extracurricular activities of sports type. In other perceptual-motor areas, there are no significant differences.

**Table 5:** Mann–Whitney-test and descriptive statistics according to the practice of sports activities on the perceptual-motor aspects.

	**Mann–Whitney-test**					**Descriptive statistics**				
**Var**	***U***	***Z***	***p-*value**	***R***	**Practice**	**Mean (SD)**	**Me**	**Range**	**G1**	**G2**
RC	50,456.5	−0.528	0.597	0.020	No	13.23 (2.31)	15	342.10	−1.167	0.408
					Yes	13.08 (2.31)	14	334.23	−1.285	1.174
BI	49,840.0	−0.908	0.364	0.035	No	19.21 (1.61)	20	344.70	−3.508	17.489
					Yes	18.82 (2.59)	20	332.81	0.163	−1.651
MD	51,644.0	−0.010	0.992	0.000	No	13.59 (1.78)	14	337.09	−1.124	0.315
					Yes	13.54 (3.92)	14	336.95	−1.991	4.851
VMC	47,648.0	−1.682	0.093	0.036	No	26.04 (3.74)	27	320.05	−1.349	2.762
					Yes	26.46 (3.64)	27	346.22	−0.941	3.564
SO	45,550.5	−2.859	0.004	0.112 (1)	No	9.14 (1.11)	10	311.20	−1.228	1.073
					Yes	9.34 (1.50)	10	351.03	1.395	40.603
SUM	50,703.5	−0.401	0.689	0.015	No	81.22 (8.66)	84	332.94	−1.265	−1.665
					Yes	81.24 (9.66)	83	339.21	1.696	6.345

*Significant differences p < 0.05*.

*SD, standard deviation; Me, Median; G1, skewness; G2, kurtosis; RC, respitatory control; BI, body image; MD, motor dissociation; VMC, visuo motor coordination; SO, spatial orientation; SUM, sum of all items*.

Table 6 presents the results in the affective-relational aspects. As can be seen in the table, there are no significant differences in these aspects.

**Table 6:** Mann–Whitney-test and descriptive statistics according to the practice of sports activities on the affective-relational aspects.

	**Mann–Whitney test**					**Descriptive statistics**				
	***U***	***Z***	***p-*value**	***R***	**Practice**	**Mean (DT)**	**Me**	**Range**	**G1**	**G2**
EC	50,059.0	−0.673	0.501	0.026	Yes	25.79 (4.19)	27	330.22	−1.339	2.081
					No	26.46 (3.53)	27	340.69	−1.055	4.524
SR	48,370.5	−1.382	0.167	0.054	Yes	20.67 (2.67)	21	350.91	−0.520	0.208
					No	20.51 (2.98)	21	329.44	−0.204	7.240
SUM	51,140.5	−0.219	0.827	0.000	Yes	46.46 (6.30)	48	339.22	−1.073	1.180
					No	46.97 (5.93)	47	335.79	−0.632	7.302

*Significant differences p < 0.05*.

*SD, standard deviation; Me, Median; G1, skewness; G2, kurtosis; EC, emotional control; SC, social relation; SUM, sum of all items*.

### Differences in Children With and Without Siblings

Table 7 presents the differences according to the presence of siblings in the family for physical-motor variables, showing that there are no significant differences between children who have siblings and those who do not have them in any physical-motor variable.

**Table 7:** Mann–Whitney-test and descriptive statistics on physical-motor variables.

	**Mann–Whitney-test**					**Descriptive statistics**				
**Var**	***U***	***Z***	***p-*value**	***r***	**Siblings**	**Mean (SD)**	**Me**	**Range**	**G1**	**G2**
LT	31,188.0	−1.797	0.072	0.167	Yes	26.25 (5.03)	24	311.02	0.872	−0.669
					No	27.00 (4.95)	26	345.40	0.043	−1.038
DC	33,403.5	−0.679	0.497	0.044	Yes	27.51 (2.65)	28	349.39	−1.233	1.348
					No	26.98 (3.38)	28	336.62	−1.150	0.858
ME	31,723.0	−1.628	0.103	0.019	Yes	14.03 (1.76)	14.5	362.73	−1.792	3.909
					No	13.72 (1.69)	14	333.57	−1.626	2.945
TPC	31,422.5	−1.714	0.087	0.139	Yes	13.26 (1.89)	13	365.12	−1.024	0.127
					No	12.87 (2.19)	14	333.03	−1.000	0.915
BAL	33,059.0	−0.857	0.391	0.000	Yes	21.81 (3.02)	22	352.13	−0.527	−0.982
					No	21.39 (3.62)	22	336.00	−0.832	0.336
SUM	33,346.0	−0.691	0.490	0.075	Yes	102.85 (10.01)	103	349.85	−0.832	−0.986
					No	101.95 (10.99)	103	336.52	0.336	1.789

*Significant differences p < 0.05*.

*SD, standard deviation; Me, Median; G1, skewness; G2, kurtosis; LT, laterality; DC, dynamic coordination; ME, motor execution; TPC, tonic-postural control; BAL, balance; SUM, sum of all items*.

Table 8 shows the results of the Mann–Whitney-test to make differences between children who have siblings and those who do not have them in the perceptual-motor aspects. There are significant differences in spatial orientation, being favorable to the group that has no siblings. In other perceptual-motor areas, there are no significant differences. Finally, Table 9 presents the results in the affective-relational aspects. There are significant differences in social relationships (higher score ranges for children who have siblings).

**Table 8:** Mann–Whitney-test and descriptive statistics on perceptual-motor variables.

	**Mann–Whitney-test**					**Descriptive statistics**				
**Var**	***U***	***Z***	***p-*value**	***r***	**Siblings**	**Mean (SD)**	**Me**	**Range**	**G1**	**G2**
RC	32,480.0	−1.443	0.149	0.020	Yes	13.45 (2.31)	14	356.72	−1.159	0.394
					No	13.06 (2.31)	14	334.95	−1.218	0.811
BI	30,530.5	–.742	0.458	0.035	Yes	19.46 (1.14)	20	372.19	−2.597	6.995
					No	18.84 (2.47)	20	331.41	−1.837	11.139
MD	33,045.5	−1.315	0.188	0.000	Yes	13.80 (1.44)	14	352.23	−1.209	0.878
					No	13.51 (2.00)	14	335.97	−1.746	3.556
VMC	32,133.0	–.899	0.369	0.036	Yes	26.78 (2.93)	27	359.48	−1.164	2.184
					No	26.22 (3.82)	27	334.32	−1.040	3.149
SO	33,423.0	−2.534	0.011	0.112 (1)	Yes	9.22 (1.01)	10	328.76	−1.128	0.487
					No	9.28 (1.45)	10	341.34	1.128	37.491
SUM	31,864.5	−1.190	0.234	0.015	Yes	82.72 (6.39)	84	361.61	−1.334	2.278
					No	80.91 (9.82)	83	333.83	−1.512	4.759

*Significant differences p < 0.05*.

*SD, standard deviation; Me, Median; G1, skewness; G2, kurtosis; RC, respitatory control; BI, body image; MD, motor dissociation; VMC, visuo motor coordination; SO, spatial orientation; SUM, sum of all items*.

**Table 9:** Mann–Whitney-test and descriptive statistics on affective-relational variables.

	**Mann–Whitney-test**					**Descriptive statistics**				
	***U***	***Z***	***p-*value**	***r***	**Siblings**	**Mean (DT)**	**Me**	**Range**	**G1**	**G2**
CE	33,548.5	−0.593	0.553	0.023	Yes	26.37 (3.68)	27	348.24	−1.753	4.369
					No	26.20 (3.81)	27	336.89	−1.128	3.428
RS	29,249.5	−2.787	0.005	0.110 (1)	Yes	21.16 (2.53)	21	382.36	−0.442	0.157
					No	20.43 (2.93)	21	329.08	−0.249	6.197
SUM	31,040.5	−1.860	0.063	0.073	Yes	47.53 (5.66)	49	368.15	−1.357	3.121
					No	46.63 (6.14	47	332.33	−0.706	5.226

*Significant differences p < 0.05*.

*SD, standard deviation; Me, Median; G1, skewness; G2, kurtosis; EC, emotional control; SC, social relation; SUM, sum of all items*.

## Discussion

Many authors have investigated on ESAs, for example, Calero et al. (29), Delgado-Lobete and Montes-Montes (30), Pérez and Geidel (31), and Park et al. (32). In addition to those studies, this research proposes a global information based on the psychomotor development and the affective relational implications on this type of activities on students who have or do not have siblings. At the motor level, specifically, children have differences in laterality and postural tonic control, with better average children practicing ESAs; spatial orientation is another aspect significantly developed in children who participate in ESAs and do not have siblings. Regarding relational affective skills, this area is much nurtured by the ESAs and by the children who have siblings.

There is a belief that children who have siblings are more developed at the motor level than those who do not have siblings; however, this research determines that there are no significant differences in most of the motor physical and perceptive variables. Regarding spatial orientation, children who do not have siblings have greater stimulation in this area, since from an early age they are forced to independently explore the environment in which they are located in, which leads to greater motor development skills, unlike children who have siblings, who can learn by imitation, but will not necessarily have greater motor skills. Space orientation brings great cognitive benefits, as it is the basis for the acquisition of reading, writing, and calculation as corroborated by studies (11, 23, 24, 26), with children without siblings being more benefited academically than those with siblings. Downey (33) highlighted in his research that cognitive stimulation is lower for children who have siblings, as the resources of the parents are diluted as the family grows.

In this research, it is recognized that ESAs provide a great benefit in children who have siblings or not, being doubly benefited at the relational affective level those who have siblings, and this result coincides with those found by authors such as Vera (34) or Villalobos and Mondragón (35). The last authors conducted a comparative study of the social skills of only children and other children. They found a lower self-concept in only children. In turn, Kitzmann et al. (36) highlighted as a result that only children are less accepted by their class group, being victimized and bullied by groups of classmates, suggesting that those with siblings better manage the conflict. Considering that these characteristics are closely related to social skills, it is affirmed that the ESAs are beneficial for only children, taking into account the outcome of this study and the other research mentioned above, since coexistence, sharing, follow-up of instructions, and collaborative learning that occurs in sports permit the children to improve their emotional development. On the other hand, possessing a habit and a recreational physical intention in which the children can use their physical skills may also strengthen their emotional development.

These results confirm, as do the research of Eime et al. (37), Fredricks and Eccles (38), Dimech and Seiler (39), or Vella et al. (40), that sports activities are beneficial to children during their development.

## Conclusions, Limitations, and Future Studies

At the motor level, it is once again demonstrated that ESAs are beneficial for the child population. In addition, spatial orientation is an area that brings benefits that are related to cognitive development especially at those early ages in the acquisition of basic instrumental reading subjects, writing, and calculation. It is also appreciated that there are no differences between children who have siblings and those who do not at the level of laterality, postural tonic, and perceptual aspects—motor.

At the affective-relational level, it is of great benefit to all children the ESAs but more for those who do not have siblings, since it provides them with a space in which they can establish social relationships, which can sometimes deteriorate within the school, giving it a second opportunity to develop in the affective development and in the establishment of social relationships, improving their community skills, reducing anxiety and psychological difficulties, and acquiring skills for better conflict management, among others.

At the level of educational and community policies, it is necessary for communities to facilitate and promote within educational institutions, or sports institutions, access to ESAs and in this way promote a better society, stimulating the parents of only children to get into these activities, exposing the benefits to them, and providing aid plans and transfer, among others, to parents of large families who, seeing their abilities diminished, deprive children of the participation in ESAs.

Some of the limitations found in this research are that the sample is not probabilistic and not generalizable to the Spanish population; however, Castilla la Mancha region is in central Spain and can be a good reflection of the country's behavior. For future studies, it should be interesting to analyze in-depth the effects of an interventional program to see the extent to which space orientation affects the psychomotor development in both children who are only children and those with siblings. Since space orientation plays an important role in acquiring knowledge of other instrumental materials, as well as improving the affective and relational dominance of children, the intervention program may be directed to improve the spatial orientation skills in both groups.

## Data Availability Statement

The original contributions presented in the study are included in the article/supplementary material, further inquiries can be directed to the corresponding authors.

## Ethics Statement

The studies involving human participants were reviewed and approved by School Ethics and Conviction Committee CRA CALAR DEL MUNDO, Albacete, Spain. Written informed consent to participate in this study was provided by the participants' legal guardian/next of kin.

## Author Contributions

PG-M: research design, information gathering, and report preparation. SR-M: data analysis and report preparation. CR-F: review of the bibliography and preparation of the report. All authors contributed to the article and approved the submitted version.

## Conflict of Interest

The authors declare that the research was conducted in the absence of any commercial or financial relationships that could be construed as a potential conflict of interest.
